# Engineered microbial consortia: strategies and applications

**DOI:** 10.1186/s12934-021-01699-9

**Published:** 2021-11-16

**Authors:** Katherine E. Duncker, Zachary A. Holmes, Lingchong You

**Affiliations:** Department of Biomedical Engineering, Duke University, Durham, NC 27705 USA

## Abstract

Many applications of microbial synthetic biology, such as metabolic engineering and biocomputing, are increasing in design complexity. Implementing complex tasks in single populations can be a challenge because large genetic circuits can be burdensome and difficult to optimize. To overcome these limitations, microbial consortia can be engineered to distribute complex tasks among multiple populations. Recent studies have made substantial progress in programming microbial consortia for both basic understanding and potential applications. Microbial consortia have been designed through diverse strategies, including programming mutualistic interactions, using programmed population control to prevent overgrowth of individual populations, and spatial segregation to reduce competition. Here, we highlight the role of microbial consortia in the advances of metabolic engineering, biofilm production for engineered living materials, biocomputing, and biosensing. Additionally, we discuss the challenges for future research in microbial consortia.

## Introduction

Over the past two decades, synthetic biologists have engineered microbes to accomplish specific tasks for various applications, including biosensing [1–4], biocomputing [5–8], and biomanufacturing [9–13]. This progress has benefited from and contributed to the development of a growing toolbox of biological components and devices [14–16].

**Fig. 1:**
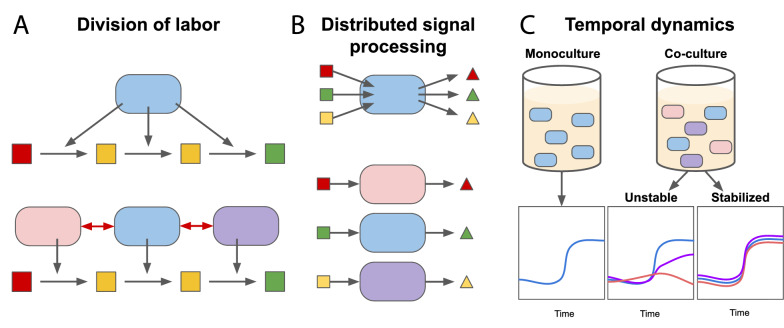
Engineering microbial consortia compared to single strains. **A** In monoculture, a single engineered strain expresses all enzymes to carry out an entire metabolic pathway. Engineering a microbial community enables division of labor, where each strain expresses one enzyme for the pathway. Intercellular communication (red arrows) may also be synthetically introduced. **B** Signal processing for biosensing or biocomputing. In a single strain, all genetic circuits are implemented in the same strain. In a consortium, the signalling circuits are distributed across populations of the community. **C** In monoculture, the culture is homogeneous so there will be no competition or interactions modifying the growth dynamics of the population (left). A challenge in consortia engineering is maintaining a stable co-culture due to interactions between strains. Often, certain strains of a microbial community dominate the environment leading other strains to extinction (middle), unless the community is engineered to account for this (right)

Typically, a single microbial population is modified with genetic circuits to carry out assigned tasks in a monoculture environment. In this case, one strain carries all the circuit components required for the overall function, such as a metabolic activity (Fig. 1A) or sensing and actuation (Fig. 1B). The engineering task becomes increasingly challenging as the circuit complexity increases. Expressing multiple circuit components can impose a significant burden on the host cell, which can drastically impact circuit dynamics or reduce overall productivity of the engineered pathway [17–19]. Crosstalk, unintended interactions between signaling components, must also be considered when designing complex circuits in cells [20–22]. Zhang et al. demonstrated another challenge of implementing synthetic circuits in a single strain [23]. They found that independent circuit components compete for gene expression resources, and this competition results in unintended correlation between different genes on the same plasmid [23].

One strategy to address these challenges is to engineer circuit functions in microbial consortia, which are communities with multiple microbial populations [16, 17, 24]. Engineering consortia enables division of labor between strains, where each population performs one task as part of the overall circuit function (Fig. 1A, B). Common methods to engineer these consortia include the use of plasmids [25–27], genome integration [28], and spatial separation of populations [29, 30]. For example, when Zhang and colleagues divided their circuit between two strains, they eliminated the gene expression resource competition, which enabled the circuit to function better [23]. Division of labor can also reduce the burden experienced by each population and simplify circuit optimization [17, 31]. However, it introduces its own set of challenges. For instance, when distributing a metabolic pathway, the molecules produced in each strain need to be released into the extracellular environment in order to operate in the overall process [17], which can reduce the pathway efficiency. Also, distributing labor across a consortium requires control of the coexistence of two or more populations, which is nontrivial. Different populations in the same community may have unintended interactions that can affect the dynamics of the consortia [25, 32]. For example, in the absence of a stabilizing mechanism, a fast-growing population can drive a slow-growing population to extinction [26] (Fig. 1C). Given these challenges and disadvantages, engineering microbial consortia is not guaranteed to be a better solution than engineering a single strain. A choice between microbial consortia or single strain engineering needs to be made depending on the design goal or application and the described benefits and challenges of each method.

Engineering microbial consortia often utilizes intercellular communication modes, such as quorum sensing (QS) [27, 33, 34] and bacteriocin expression [25], to mitigate this competition between strains or coordinate gene expression. Here, we discuss recent progress in engineering stable microbial consortia and their potential applications, as well as remaining challenges for future research.

## Programming microbial consortia interactions

**Fig. 2:**
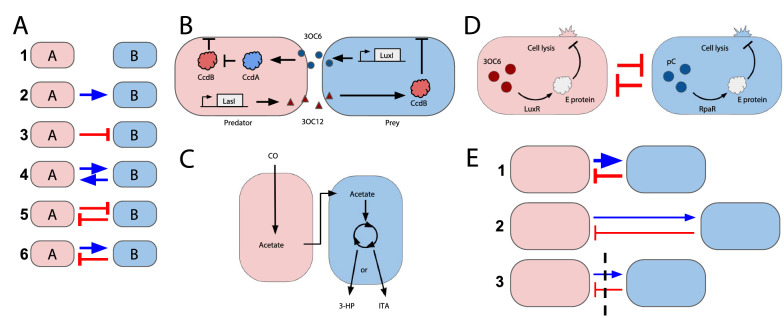
Engineering interactions in microbial consortia. **A** There are six possible pairwise interactions between two strains. These consist of no interactions (1: neutralism), one-way interactions (2: commensalism and 3: amensalism), and two-way interactions (4: mutualism, 5: competition, and 6: predation). Adapted from Kong et al*.* [25]. (1) Neutralism: Both populations exist with no impact on the other. (2) Commensalism: B benefits from A (i.e., the growth of B is promoted by the presence of A), but A is not affected by B. (3) Amensalism: B is inhibited by A, but A is not affected by B. (4) Mutualism: A and B are mutually beneficial. (5) Competition: A and B mutually inhibit each other. (6) Predation: B benefits from and inhibits A. **B** Predator strain constitutively expresses toxic ccdB which can be suppressed by ccdA that is induced by QS signal 3OC6HSL expressed by the prey strain using the lux system. The predator strain expresses QS molecule 3OC12HSL using the las system that induces toxic ccdB expression in the prey strain. Adapted from Balagadde et al*.* [27]. **C** *E. limosum* uses a native metabolic pathway to convert CO into toxic acetate. *E. coli* are engineered to intake acetate and convert it to itaconic acid (ITA) or 3-Hydroxypropionic acid (3-HP). Adapted from Cha et al*.* [44]. **D** Two strains expressing orthogonal synchronized lysis circuits. Both strains use quorum sensing to induce cell lysis. They also have competition due to limited space. Scott et al. found the LuxR and RapR quorum-sensing systems could be used orthogonally. Lysis will only be induced in a strain if its quorum-sensing molecule is at a high enough concentration. The LuxR system senses 3-oxo-C6 HSL (3OC6), and the RpaR system senses p-Coumaroyl HSL (pC). This resulted in the two strains coexisting despite different growth rates. Adapted from Scott et al*.* [26]. **E** (1) A microbial consortium in a well-mixed culture can have interactions between different populations. Spatial arrangement can decrease the strength of these interactions by controlling (2) the distance between different populations on a surface or (3) separating different populations with a physical barrier. Arrow thickness represents strength of interactions

A pair of populations, A and B, can have one of six types of ecological interactions: commensalism, amensalism, mutualism, competition, predation, and neutralism (Fig. 2A) [29]. As a microbial community increases in the number of different populations, the community complexity increases due to the combinatorial increase in the number of pairwise interactions and emergence of higher order interactions [35]. For instance, the presence of a third population can change the strength or the type of a pairwise interaction, and a fourth population can then change that interaction [36]. The stability of a community can depend on the types and strengths of these interactions, and computational modeling can be used to predict the community composition over time [36–38]. Synthetic microbial consortia can be designed using pairwise interactions, but there is a need for faster and scalable development of these interactions [25]. The following section highlights some of the implementations using ecological interactions to construct microbial consortia.

### Predator–prey

An early example of a synthetic microbial community engineered to exhibit a two-way interaction via QS is the predator–prey system developed by Balagadde et al. [27]. Two *Escherichia coli* populations, a predator strain and a prey strain, were engineered to communicate through QS molecules (Fig. 2B). The predator constitutively expresses a suicide protein (CcdB). The prey benefits the predator by generating QS molecules that activate the predator’s expression of an antidote protein (ccdA) to suppress the predator’s CcdB expression. When the prey cell density is low, the predator will start to die due to the lack of antidote [27]. Additionally, the predator produces a QS molecule that induces CcdB expression in the prey [27]. When the predator cell density is low, the prey cell density increases due to less suicide protein expression [27]. The authors developed a mathematical model to predict conditions under which the system generates three different population dynamics: (1) prey domination, (2) oscillations between predator and prey, and (3) predator domination. They validated this model by demonstrating each type of behavior experimentally, where the dynamics in each experiment were controlled by varying inducer concentration [27].

Since then, many synthetic ecosystems have been constructed with additional strains or more complex interactions. A similar predator–prey ecosystem was designed where a prey strain deactivates antibiotics that target the predator, effectively protecting the predator [39]. Meanwhile the predator expresses a bacteriocin that kills the prey. Additionally, an invader strain was introduced that kills both predator and prey. When the invader was designed to benefit from the prey’s antibiotic deactivation and to be killed by the predator’s bacteriocin, the predator and prey showed the same oscillatory behavior as without the invader, while the invader temporarily grew then died out. When the invader was designed to be killed by the predator but not benefit from the prey, both the predator and prey die out and the invader grows and thrives. These examples of modeling microbial interactions and implementing communication through quorum sensing, antibiotics, and gene circuit components serve to help understand microbial interactions in nature and predict behaviors of more complex synthetic consortia.

### Mutualism

Mutualistic interactions are ubiquitous in natural communities. Synthetic microbial communities have been engineered to provide quantitative insights into the persistence and function of mutualistic systems [40–43]. Moreover, mutualistic interactions have been utilized to improve biofilm formation and metabolic production [24, 44, 45]. For example, a synthetic microbial consortium consisting of an *E. coli* strain engineered to help a second engineered *E. coli* strain form a biofilm was mutualistic due to the reliance of both strains on biofilm formation for optimal growth or survival [45].

Synthetic consortia have been designed with mutualism to improve metabolic engineering for producing valuable metabolites [24, 44]. Zhou et al*.* constructed a mutualistic microbial consortium where *E. coli* excretes acetate, which inhibits its growth; *Saccharomyces cerevisiae* uses acetate as its sole carbon source in the culturing environment, which effectively reduces the acetate concentration, allowing *E. coli* to survive. By exploiting the advantages of each species, this study divided a metabolic pathway to produce taxanes between the mutualistic *E. coli* and yeast strains. The mutualistic design improved the stability of the co-culture composition, increased product titer, and decreased variability in product titer compared to competitive co-cultures [24].

Similarly, Cha et al. designed a mutualistic co-culture to improve metabolic conversion of carbon monoxide (CO). In particular, *Eubacterium limosum* naturally consumes CO as a carbon source and converts it to acetate, which negatively affects cells when it accumulates [44]. *E. coli* cells were engineered to convert acetate in the mixed culture into a useful biochemical, either itaconic acid or 3-hydroxypropionic acid [44] (Fig. 2C). They demonstrated more efficient CO consumption and biochemical production in the engineered mutualistic consortia than in *E. limosum* monoculture [44]. This mutualistic design represents a platform where adding a mutualistic engineered strain can improve and exploit natural metabolic processes, like CO consumption, carried out by microbes that are difficult to engineer genetically.

### Implementing negative feedback to mitigate competition

In the absence of mitigating mechanisms, two strains competing for the same nutrient and space in a co-culture cannot stably coexist unless they have the same growth rate. The faster-growing strain will eventually exclude the slower-growing strain. One approach to mitigate the competition is to impose negative feedback on each population through programmed population control [46].

Scott et al. demonstrated this strategy by using two orthogonal synchronized lysis circuits (SLC) to generate a stable co-culture of two engineered E. coli populations (Fig. 2D) [26]. Each SLC uses QS molecules to induce lysis of the host population once the population increases to a sufficiently high density, which implements negative feedback. As a result, the faster-growing strain starts lysing upon reaching a high density, and this reduces its inhibition on the slower population (through competition). Once this lysis occurs, the slower-growing strain is able to grow to a higher density until it lyses as well. By introducing these negative feedback loops, each strain is self-limiting, which offsets the effects of competition and allows the strains to coexist.

### Programming consortia based on pairwise interactions

Studies have demonstrated that well-defined pairwise interactions can enable predictable programming of more complex communities. In other words, in the communities studied, the contribution of higher-order interactions is not critical for the prediction of the overall community dynamics. Kong et al*.* designed all six pairwise interactions into synthetic microbial consortia [25]. The interactions were designed by introducing gene circuits with beneficial or detrimental effects on the other population. For example, in their commensalistic pair, one strain secretes the signalling molecule nisin, which induces tetracycline resistance in the second strain. Also, they designed a competitive pair by engineering each strain to produce a toxin that kills the other strain. From their initial experiments, Kong et al. then designed three and four strain populations of synthetic microbial consortia by controlling the pairwise interactions between each strain. They used models to guide their consortia design, and they successfully implemented stable consortia with a variety of pairwise interactions.

Likewise, Friedman et al*.* assessed the predictive power of these interactions by using both computational and experimental frameworks for an eight-member community [47]. First, they experimentally characterized all the pairwise interactions between the strains to determine if they could coexist or if one strain would exclude the second. Using these results, they computationally predicted the behavior of all possible three-member communities. Upon experimental validation of the three-member communities, they found their model correctly predicted the final composition of 89.5% of the consortia. Next, they used the results from their three-member consortia to build a predictive model for the composition in seven and eight member communities. By accounting for the three-way interactions, they were able to build a model which correctly predicted 86% of the final consortia composition, whereas using only the pairwise interactions in the model resulted in a 62% accuracy. This study demonstrates the need for higher-order interactions in the development of computational models for microbial consortia. Similarly, in studying the impact of bacteria contaminants in yeast production of bioethanol, researchers found that using only pairwise interactions to develop a model led to large differences between the model and experiments [48]. They concluded that the unaccounted-for higher-order interactions are responsible for stabilizing communities with more than two species [48].

Similarly, Venturelli et al*.* developed a model to predict the final composition of a consortia of gut microbiota [49]. Their findings also indicate that inclusion of pairwise interactions into their model resulted in higher overall accuracy in predicting more complex consortia composition. One unique aspect of their study was the analysis of “history dependence” on the outcome of the composition. They initially tested the pairwise interactions between strains by seeding each pair in a 1:1 ratio, and then measuring the change in population composition over time. Next, they performed the same experiments but tested initial seedings of 1:19 and 19:1. They found that the initial seeding quantity impacts the pairwise interactions because the final steady state compositions were different depending on the initial seeding quantity in these pairwise experiments. Using these results, they expanded their model to show that this history dependence is a function of how strong the interactions are between the strains. Depending on the interaction strength, they can predict whether or not there will be history dependence in the pairwise interaction.

### Using signalling to control consortium gene expression

By design, both the engineering and overall function of a microbial community are tied to coordinated dynamics in and between constituent populations, which is often mediated by the synthesis and sensing of small, diffusible signaling molecules. These interactions between individual populations can be engineered to measure community level dynamics. For example, a synthetic consortium was designed with two communicating populations that only express their respective signals when both populations are at high cell densities [50].

Chen et al*.* designed a synthetic consortium that generates synchronized oscillations in gene expression in two strains using QS molecules. The QS molecule produced by an activator strain upregulates fluorescent protein expression in both strains while the QS molecule produced by a repressor strain down-regulates the fluorescent protein expression in both strains [34]. This design along with a negative feedback loop produced robust coordinated oscillations of gene expression in the two populations [34].

Alnahhas et al. developed two-population microbial consortia which could sense which strain was in the majority, and the majority strain would express its fluorescent protein [51]. These consortia functioned using corepressive circuits, such that each population repressed the expression of the other’s quorum-sensing molecules. This resulted in whichever strain made up the majority of the consortia being the only strain to express its fluorescent protein. In designing these consortia, the authors controlled the composition of the populations and demonstrated functional sensing of the composition.

### Spatial partitioning and arrangement to modulate interactions

While engineering ecological interactions between strains is one way to achieve stable coexistence [24–26], another method to control consortia stability without the need to engineer genetic circuits is through spatial separation [29, 30]. Kim et al*.* found that a microbial community can be stabilized, despite differing growth rates, by spatially separating the subpopulations to an optimized distance [52]. In a well-mixed culture of three mutualistic bacterial species that cannot survive individually, one population grew rapidly while the other two decreased in cell density over time [52]. However, when each population was physically separated but still able to chemically communicate with one another, all three had stable growth [52]. At increased distances though, the cell density of all three species declined, indicating that populations need to be close enough to benefit from positive interactions but separated enough to minimize growth competition [52]. Spatial arrangement of microbes modulates both competitive and positive interactions between different populations of a consortium by modifying the rate that communication molecules are exchanged between strains via distance or physical barriers (Fig. 2E) [52].

Johnston et al*.* used hydrogels to spatially separate two different organisms in a commensalistic microbial co-culture of engineered *E. coli* and yeast for biomanufacturing of betaxanthins. *E. coli* were engineered to produce L-dopa, and *S. cerevisiae* were engineered to convert L-dopa to betaxanthins [29]. No other ecological interactions were engineered through genetic circuits [29]. Culturing each strain in separate hydrogel structures increased betaxanthin production and decreased variability between samples, compared to mixed consortia [29]. The spatial separation also enables the final composition to be controlled by changing the mass ratio of gels added to the culture [29]. The hydrogels provided an additional advantage of enabling repeated use and protection for long term preservation of the consortia [29].

Similarly, Dai et al. spatially separated *E. coli* populations in polymeric microcapsules to control the consortia composition for the division of labor of a multienzyme metabolic pathway for fatty acid synthesis [53]. Controlling protein ratios is important for complex extracellular bioproduction pathways [31]. While expressing and purifying proteins in a one-pot culture is cheaper than individually culturing and purifying each one separately, protein ratio control has been a challenge in these mixed cultures [31]. To address this limitation, Dai et al*.* spatially separated strains in a one-pot culture to control strain composition and thereby control protein output ratios directly by the number of capsules of each strain added to the culture [53]. Using this design platform, they successfully constructed fatty acids with seven enzymes expressed in seven different strains each separated by the polymeric microcapsules in the same liquid culture [53]**.**

Beyond spatially separating strains for composition control and higher product output, synthetic microbial consortia can be spatially arranged in specific structures for gene expression control, patterning, and observing cell signaling [16, 54, 55]. Despite providing higher composition stability, increased distance results in weaker interpopulation signaling because the signal molecules need to diffuse further distances [56]. However, gene expression can be coordinated among spatially distant populations of a consortia through engineered circuits [57]. Gupta et al. utilized spatial separation and distance as a tool to tune gene expression. They developed a microfluidic platform to control the distance between populations that could only interact through diffusible molecules [54]. This demonstrated that increased distance improves the reliability of oscillatory signal transmission between cells by reducing noise, but it also decreases the magnitude of gene expression induced by the signal [54]. Tei et al*.* used a silicone mold in solid media to construct compartments and channels for cells to be physically separated and AHL molecules to flow between. By arranging a two-strain consortia with cross-repressive gene expression, they found that changing the number of connecting channels from the center played a role in generating contrasting patterns in gene expression between the two strains [55]. This demonstrates that spatial control of populations can have a more direct effect on interactions than biochemical parameters [55].

## Applications

A major reason for engineering microbial consortia is to achieve complex processes. Synthetic biologists are shifting from proof-of-concept consortia design to using consortia for specific applications. In recent years, scientists have engineered microbial consortia to improve the yield of metabolic pathways [58], achieve distributed production of multiple protein products [31], increase biofilm production in *Bacillus subtilis* communities [28], and implement logic gates using orthogonal bacterial signaling pathways [16]. These successes were possible due to division of labor, and each used multiple populations of bacteria to coordinate an overall function.

In general, it becomes more advantageous to use a microbial community with division of labor to carry out a metabolic pathway when burden, toxicity, complexity, or number of steps increase [17]. Conversely, using a single population can be more efficient for simpler metabolic pathways with less burdensome or toxic components. To quantify this reasoning, Tsoi et al*.* developed a mathematical model to determine criteria for when division of labor would be more efficient than monocultures for metabolic pathways. This logic can further be applied to biofilm production, biocomputing, and biosensing.

### Metabolic pathways

Metabolic engineering aims to optimize production of metabolites and biomolecules using engineered cells. Research in metabolic engineering has primarily involved optimizing single strains [9, 10]. There is growing research to develop microbial consortia for metabolic engineering to divide genetic circuits for the pathways between strains [24, 44, 58–61]. More steps of a metabolic pathway require more enzymes to be expressed, which can increase the burden on cells in a monoculture [17]. Distributing the steps of the metabolic pathway between multiple microbial populations in a culture can be advantageous for these more complex pathways [17]. When using multiple populations, the metabolites must transport effectively between different populations, which may mean passive diffusion, active transport, or reactions occurring in the extracellular space [17]. To this end, engineers have built specialized transporters [62, 63] and colocalized enzymes to maximize production [64, 65]**.** Due to these transport requirements, some pathways are better suited for single strains.

**Fig. 3:**
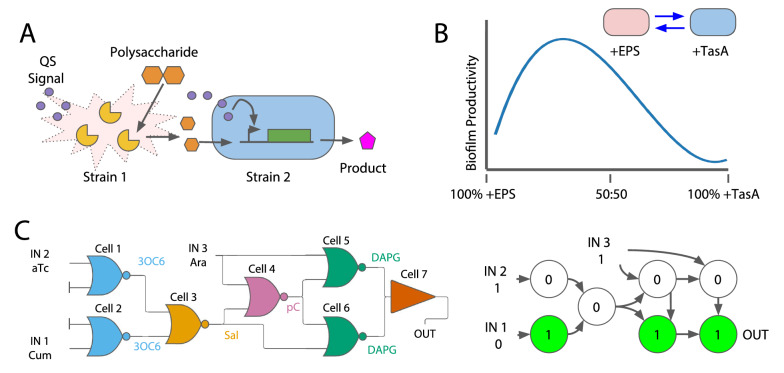
Applications of engineered microbial consortia. **A** Example of a two-strain microbial consortium for metabolic pathway engineering. Strain one breaks down polysaccharides and lyses to release the monosaccharides and QS signals which induce conversion of the monosaccharide to a final product. Adapted from Honjo et al*.* [59]. **B** Schematic of biofilm productivity as a function of starting ratio between strains expressing EPS or TasA. The biofilm productivity can be tuned by using different starting ratios. At its maximum, the synthetic consortium biofilm productivity is greater than the wild-type consortium. Adapted from Dragoš et al*.* [28]. **C** An implementation of an AND-XOR logic gate. This implementation uses 7 cells and 4 communication signals. Cells 1 through 6 are NOR gates, so if a signal molecule is present they will be OFF. Cell 7 is a buffer, so either signal from cell 5 or 6 is shown as the output. To the right of the logic gate is a schematic showing the implementation of one set of inputs for the system. Adapted from Du et al*.* [16]

Honjo et al*.* developed a synthetic microbial consortium composed of different strains designed to sequentially carry out steps of a metabolic pathway processing cellobiose into isopropanol [59]. Two *E. coli* strains were constructed using gene circuits: one produces the enzyme to break down polysaccharides from cellobiose and the other strain converts processed polysaccharides into isopropanol. The enzyme-producing strain also expresses QS molecules to induce self-lysis at high cell density. Lysis releases the enzymes to process the polysaccharides. The QS molecules also induce the isopropanol production pathway in the second strain to convert the processed polysaccharides into isopropanol (Fig. 3A).

For effective implementation of complex metabolic pathways, multiple enzymes need to be expressed at balanced ratios to optimize productivity [31, 53, 58, 66]. Enzyme ratios in microbial consortia can be controlled by population composition [31]. Stable coexistence of multiple populations has been achieved through synthetic gene circuits and spatial arrangement [24–27, 52]. Furthermore, synthetic biologists are developing tools for more precisely tuning the final composition of microbial consortia [29, 51, 53].

A method to control population composition is to inoculate the populations of a synthetic consortium at specific initial ratios [58]. However, the relative ratios can change over time due to interactions and varying growth dynamics of the strains. Dinh et al*.* addressed this limitation by utilizing a QS circuit to regulate growth of a strain over time in co-culture as a method to control final strain composition for naringenin production [58]. The growth control circuit uses QS molecules produced by a strain that accumulate as it grows to downregulate its growth rate at higher cell densities. When implementing the growth control circuit in a co-culture designed to carry out the naringenin production pathway, the naringenin titer was higher than both monoculture and co-culture without the growth control.

Another method to control population composition is through spatial arrangement. Shahab et al*.* created modular synthetic microbial consortia using an oxygen gradient to spatially arrange different populations [67]. Using a membrane-aerated reactor, they cultured three different strains to convert cellulose and xylose into short-chain fatty acids [67]. The aerobic *Trichoderma ressei* provided cellulolytic enzymes to break down cellulose and xylose, the facultative anaerobic bacteria *Lactobacillus pentosus* converts the intermediate sized sugars to lactate, and the anaerobic *Clostridium tyrobutyricum* converts lactate into butyric acid [67]. Due to their differences in oxygen usage, they exist in a gradient from high oxygen concentration at the membrane to low concentration furthest from the membrane in the reactor. This gradient allows for the coexistence of different populations. To demonstrate modularity, they exchanged *C. tyrobutyricum* for other anaerobes to produce other short-chain fatty acids including acetic, propionic, valeric, and caproic acids [67]. These researchers took advantage of natural metabolic pathways and oxygen requirements to engineer a modular system capable of converting biomass into relevant chemicals.

### Biofilm productivity

Synthetic biologists are engineering materials as living systems because living systems can evolve, self-organize, and respond to the environment [68]. One common strategy to design engineered living materials (ELMs) is to use biofilm-forming bacteria to form a structured material because biofilms can be controlled via protein and genetic engineering of cells [69]. In natural systems, bacteria form biofilms to colonize plants and form symbiotic relationships [70] or to provide antibiotic resistance on wound infections [71]. Biofilms are formed by many organisms, including *E. coli* and *Salmonella* spp. [72], *Pseudomonas aeruginosa* [73], and *B. subtilis* [74]. Applications of engineered biofilms include manufacturing aquaplastics [75], minimizing fouling on reverse-osmosis membranes [76], and producing electricity in microbial fuel cells [77, 78]. Controlling and optimizing the production of biofilms provides a foundation for further development of ELMs.

Biofilms can be used to promote mutualism in synthetic communities, and an early example of this was shown in experiments by Brenner and Arnold [45]. Their experiments involved using two strains of bacteria. The “blue population” could produce biofilms, but repression of the gene *DapD* resulted in a strain which could not synthesize diaminopimelate and lysine. Meanwhile, the “yellow population” lacked the genes to produce biofilms, but this strain expressed butanoyl-homoserine lactone (C4HSL). The yellow population could not survive in the flow experiments alone due to its inability to produce biofilms. The C4HSL molecule from the yellow population induced *DapD* expression in the blue population, which resulted in biofilm formation. With the formation of the biofilm, both strains mutually benefited from the presence of the second strain, and their overall growth was higher when both strains were present.

Dragoš et al*.* explored different strategies using division of labor within a biofilm-forming strain of *B. subtilis* [28]. The *B. subtilis* forms an extracellular matrix by producing both exopolysaccharides (EPS) and the structural protein TasA. Their study included a natural strain with three phenotypically different subpopulations as well as an engineered consortia with two genetically different populations. In the wild type, each subpopulation has a different production requirement: (1) EPS and TasA producing, (2) EPS only producing, and (3) neither producing. These differences in the subpopulations are driven by phenotype, meaning that they all have the genotype to produce both EPS and TasA, but different expression levels drive the division of the strains.

Following this natural example, Dragoš et al*.* designed a microbial consortium consisting of two genetically different populations: (1) EPS producing and (2) TasA producing. The strict division of labor in the synthetic consortium results in higher biofilm production than the wild type when seeded with the proper ratio between the two populations (Fig. 3B).

### Biocomputing

Biocomputing is a field in which biological parts are used to complete computations. Some common examples of biocomputing include implementing logic gates in cells [79]. Recent advances include applying CRISPR/Cas9 to turn human cells into central processing units [80], using DNA origami to cryptographically secure data [81], and implementing an AND-XOR logic gate using 4 unique communication pathways [16]. While these advances encompass a broad array of biocomputing, the work by Du et al. and their design of the AND-XOR gate relies directly on the use of microbial consortia. This work builds on previous examples of developing distributed biocomputing systems, such as using quorum-sensing to “wire” signals between two different strains [5] or implementing a 1-bit adder in yeast populations [6].

Biocomputing is limited by the difficulty in optimizing large-scale gene circuits in a single strain [16]. An alternative is to use multiple strains to distribute the computations. To use multiple strains, the strains must communicate concurrently and orthogonally, which can be done using QS signals as “wires” between different strains [82]. Tasmir et al*.* constructed a library of all 16 two-input logic gates in *E. coli* using two orthogonal QS systems [5]. These communication channels were incorporated into cells to build OR and NOR gates, which can be combined to form the entire library. Their work demonstrated an effective way to implement orthogonal communication “wires” in multicellular logic gates. Using a similar approach, Regot et al*.* implemented logic gates in yeast using two orthogonal signalling pheromones [6]. In their work, they used these logic gates to build both a multiplexer and 1-bit adder. The multiplexer is capable of computing three inputs into one output. The 1-bit adder is the building block for a binary calculator. The creation of these computational blocks is still used to demonstrate new circuitry, as seen in a 2019 paper using human cells as CRISPR/Cas9 central processing units [80].

In the previous examples, the authors used orthogonal communication molecules to separate the biological wires between strains [5, 6]. Using a different approach, Macia et al*.* developed circuits using up to six inputs while only using one communication molecule between cells [8]. To distribute the sensing, Macia et al*.* developed a spatially separated system where they divided the computations into two layers. First, they used an input layer where cells would sense input molecules. They used six different inputs, and all of the cell types would only sense one of those inputs. Depending on whether the cells were IDENTITY or NOT gates, they would express a communication pheromone which could be sensed in the output layer. In the output layer, cells would sense whether or not the communication pheromone was present. From this, the output layer signal could be compiled into one BUFFER gate which would give a binary output of the system. The input and output layers were physically separated by using different flow chambers. This allowed the experiments to be completed using minimal engineering components: only one signalling molecule was used, and each cell had a one-input-one-output circuit. Using these modular cells, they implemented a variety of complex logic functions. In one experiment, they programmed a “3-input majority rule” system which would give a positive output if at least two of the three inputs were present. This system is important in electronics, because it functions as a redundant communication system between components to prevent failure. In another experiment, they create a “4-input comparator” which would compute whether binary signal A was less than, equal to, or greater than binary signal B. They finally demonstrated how scalable their system was by using the cells to implement a “4-to-1 multiplexer”, which had not been done previously in biological circuits. This system computed the 6 different inputs in 64 different configurations. This provides a benefit because the multiplexer is able to function as different logic gates depending on which selector inputs are given.

Du et al*.* implemented an AND-XOR gate by combining six NOR gates and one buffer gate (Fig. 3C) [16]. Each of these seven gates were implemented in an individual cell strain. The cell strains communicated using four orthogonal signalling pathways, which is the first time that four pathways have been used in one implementation. Their system relies on spatial organization to control the interactions between strains. The cellular signalling pathways work by having a sender strain express a small molecule which is sensed by a receiver strain. The pathways are implemented by placing these sender/receiver pairs in proximity, which they did by spotting *E. coli* on agar plates. This spatial separation allowed all the strains to exist without competition or other unintended interactions. After they developed the sender/receiver pairs, they implemented two-, three-, and four-channel pathways. The fluorescence patterns in these pathways showed the orthogonal signalling of each circuit: only the cells closest to the sender molecule expressed fluorescence. Finally, they built the seven-strain, four-channel AND-XOR gate (Fig. 3C). The distribution of the AND-XOR logic circuit was possible due both the orthogonality of the signalling pathways and the spatial separation of the strains. The orthogonality allowed for each computation to execute independently of the other computations. The spatial separation allowed each strain to coexist without any influence or crosstalk from the other strains.

### Biosensing

Microbes can be engineered to sense a variety of signals for applications like sensing disease biomarkers [83–85], detecting environmental contaminants [86–88], and measuring product formation in biomanufacturing [89–91]. These living biosensors are an alternative or complement to chemical or electrical based analytical methods that enable in situ*,* real-time, and low-cost sensing. Biosensors have been constructed in microbes to detect light, chemicals, biomolecules, pH, and temperature [83, 92–95]. Cells are programmed to output fluorescent or colorimetric reporters, antibiotic resistance, toxins, or functional actuators, such as therapeutics. Existing sensors are being combined and enhanced to create more complex sensing systems using single strains [96] or engineered microbial consortia.

Synthetic biologists have designed microbial consortia to control the response to a signal [97]. Shaw et al*.* increased the operational range of sensors for adenosine and melatonin when using microbial consortia compared to a single strain [97]. They combined multiple yeast populations which were engineered with different sensitivities to the respective signal and took the average readout of the consortia [97]. Increased operational range enables quantification of the respective analyte. Additionally, they used mixed populations of yeast strains to convert linear sensor responses to digital responses to the presence of melatonin or *Paracoccidioides brasiliensis* [97]. They achieved greater sensitivity than the single strain, and the digital response can be used in point-of-care applications [97].

Microbial consortia can integrate several different signals [86]. Wang et al*.* created a three-input sensor consortium that generated a fluorescent response only when arsenic, mercury, and copper were all detected [86]. One population contained transcriptional regulator sensors for arsenic and mercury, which were connected by a genetic AND gate to output a QS molecule [86]. The other population had sensors for both copper and the QS molecule produced by the first population connected by an AND gate with a fluorescent protein output [86].

Additionally, microbial consortia can improve sensing of a single target by amplifying the signal or reducing crosstalk between steps of the sensing pathway [87, 88]. Khatun et al*.* used a microbial consortium to detect organophosphorus pesticides (OPs), an environmental contaminant [87]. Cells can indirectly detect OPs by sensing *p-*nitrophenol (PNP), a product of OP hydrolysis, via a transcriptional regulatory protein [98]. Khatun et al*.* distributed the OP hydrolysis pathway and the PNP sensor-reporter circuit between two strains to eliminate interference between the two processes, thus dividing multiple steps of sensing this type of compound between strains [87].

Microbial communities enable the combination of different applications, such as metabolic engineering and biosensing. Meyer et al. created a two-population community consisting of a *B. subtilis* producer strain for vitamin B2 and an *E. coli* sensor strain to sense B2 to optimize biomanufacturing [91]. Microbial consortia provide a platform to expand the capabilities and applications of the microbial biosensing field.

## Conclusions and future prospects

Synthetic consortia have been successfully used in a variety of applications, and they have been designed both by utilizing and by minimizing their ecological interactions. Within synthetic biology, there is a push for creating a toolbox of standard parts to use in synthetic consortia design [99]. A few examples of this include using pairwise interactions to develop multi-population consortia [25], creating a cellular adhesion toolbox for different biofilm structures [100], and designing orthogonal signalling pathways for cell-to-cell communication [16].

Moving forward, there are still many challenges in the development of synthetic microbial consortia. Some areas to be improved are understanding natural complex communities, developing orthogonal cell-to-cell signalling, reducing mutants in genetically modified consortia, and controlling population composition.

Ecological interactions can be used to control the composition of a synthetic microbial consortium, and as these interactions are more tightly controlled there will be room for more complexity in these consortia. As researchers learn more from natural complex communities, such as gut or soil microbiomes, these learnings can be applied to the design of synthetic microbial consortia. Conversely, the control of engineered microbial consortia will help researchers understand the complex interactions that can happen in natural communities. For example, after Dragoš et al*.* concluded their synthetic consortia could produce biofilms similar to the wild-type, they used the synthetic consortia to colonize plant roots [28]. From this experiment, they found that the consortia behaved similarly in the plant root experiments as in the laboratory experiments.

As synthetic systems increase in size with interacting parts, there is a strong need for orthogonal signalling between cells. Much progress has been made in intercellular communication, including the implementation of logic gates in multi-strain systems [5, 6], spatial partitioning as a method to limit crosstalk [8, 30, 53], and the development of libraries of orthogonal signalling pathways [16]. This work is promising, but there is still a need for increasing the number of signals which can be used in cell-to-cell signalling. As synthetic biologists develop orthogonal signalling pathways, this can be compounded with spatial control to design more advanced logic circuits.

Another challenge in designing synthetic microbial consortia is the emergence of mutants. While strategies have been developed for managing mutants that occur, strategies to prevent their occurrence will drastically increase the robustness of synthetic microbial consortia [101]. As these underlying mechanisms are further understood, synthetic biologists will continue to design modular toolboxes to implement increasingly complex designs.

Microbial consortia provide a platform to divide the labor of metabolic pathways. An efficient metabolic pathway requires stoichiometric control of the enzymes from each population, which requires control of the ratio between different populations. There has been success in this regard, such as Dinh et al*.* using a QS circuit to decrease growth as cell density increases [58] and Dai et al*.* encapsulating cells to express enzymes for a 7-step fatty acid pathway in one culture [53]. Future challenges will require tighter control of the populations, especially as the number of populations in a culture increases. To address these challenges, engineers will continue to build on innovative work using circuits to sense the relative population density between strains [51], applying inducible signalling to control when genes are expressed [102], and spatially separating populations to control relative population ratio [53]. Additionally, metabolite transport will need to be considered in the development of these consortia.

## Data Availability

No new research data were generated for this article.

## Abbreviations

QS: Quorum sensing
CO: Carbon monoxide
SLC: Synchronized lysis circuit
C4HSL: Butanoyl-homoserine lactone
EPS: Exopolysaccharides
OP: Organophosphorus pesticide
PNP: *p-*Nitrophenol
ELM: Engineered living material
